# Anti-cyclic citrullinated peptide antibodies in primary Sjögren syndrome may be associated with non-erosive synovitis

**DOI:** 10.1186/ar2420

**Published:** 2008-05-07

**Authors:** Fabiola Atzeni, Piercarlo Sarzi-Puttini, Nicola Lama, Eleonora Bonacci, Francesca Bobbio-Pallavicini, Carlomaurizio Montecucco, Roberto Caporali

**Affiliations:** 1 Rheumatology Unit, L. Sacco Hospital, University of Milan, Via G.B. Grassi 74, 20127 Milan, Italy; 2 Department of Medicine and Public Health, Second University of Naples, Via L Armanni 75, 80139 Naples, Italy; 3 University of Pavia, IRCCS Policlinico S. Matteo, Piazzale Golgi 12, 27100 Pavia, Italy

## Abstract

**Introduction:**

The purpose of this study was to investigate the prevalence of cyclic citrullinated peptide antibodies (anti-CCP) in patients with primary Sjögren syndrome (pSS) and its correlation with clinical and laboratory data.

**Methods:**

We analysed the clinical and serological data of 155 consecutive patients with pSS. Among these, 14 were excluded due to fulfillment of American College of Rheumatology criteria for rheumatoid arthritis (RA). So, 141 patients (27 males and 114 females; mean age 48 years, range 39 to 60) were clinically assessed for the presence of synovitis (objective swelling of one or more joints) and extra-glandular involvement. The anti-CCP antibodies were tested using a commercially available second-generation enzyme-linked immunosorbent assay. IgM rheumatoid factor (RF) was determined by nephelometry.

**Results:**

Fourteen patients (9.9%) had moderate to high levels of anti-CCP, and 94 (66.7%) were positive for RF. Eighty-one (57.4%) showed extra-glandular involvement, and 44 (31.2%) had synovitis without any radiographic sign of erosion. There was a close correlation between the presence of anti-CCP and synovitis (*P *< 0.001) but no association between anti-CCP and extra-glandular involvement (*P *= 0.77). Multivariate analysis confirmed the association between anti-CCP and an increased prevalence of synovitis (prevalence odds ratio for positive versus negative anti-CCP status 7.611, 95% confidence interval 1.475 to 74.870; *P *= 0.010).

**Conclusion:**

Only a minority of patients with pSS are anti-CCP-positive, which seems to be closely associated with the prevalence of synovitis. Anti-CCP positivity in patients with pSS therefore may be a predictor of future progress to RA or an expression of the inflammatory process of synovial tissue.

## Introduction

Primary Sjögren syndrome (pSS) is a chronic, slowly progressive, inflammatory, autoimmune disease that is characterised by lymphocytic infiltration of the exocrine glands (which reduces or eliminates glandular secretion) and marked B-lymphocytic cell hyper-reactivity, which initially is manifested by a variety of serum autoantibodies, including those against Ro (SSA) and La (SSB), and rheumatoid factor (RF) [1-4].

Most patients with pSS present only with keratoconjunctivitis sicca and xerostomia, but approximately 40% develop extra-glandular musculoskeletal manifestations; lung, kidney, and skin involvement; vasculitis; neuropathy; and lymphoma [1,2]. The most common are arthralgia and an intermittent non-erosive polyarthropathy affecting mainly the small joints, which means that the picture may mimic that of rheumatoid arthritis (RA), particularly as 50% to 80% of cases are RF-positive [4,5]. However, the fact that tests for cyclic citrullinated peptide antibodies (anti-CCP) usually are negative may help to differentiate the two conditions. Anti-CCP antibodies, which were first described in 1998, were found to be highly specific in the diagnosis of RA (95%) and only slightly less sensitive than IgM RF (60% to 70%) [6,7], whereas the second generation of anti-CCP antibodies have a sensitivity of 80% and a specificity of 98%. Follow-up studies of patients with early RA have demonstrated that anti-CCP antibodies independently predict the development of erosions [8,9], but Goëb and colleagues [10] found anti-CCP autoantibodies in only 4% of 137 women and 16% of 11 men with pSS.

Gottenberg and colleagues [11] studied a cohort of 134 patients with pSS and found that 7.5% of the serum samples were positive for anti-CCP antibodies and 5.2% were positive for anti-keratin antibodies (AKAs) without any radiographic evidence of erosion after a long follow-up. They suggested that the anti-CCP-positive patients, who may be prone to developing RA, require cautious clinical and radiographic follow-up. The aims of this study were to verify the prevalence of anti-CCP antibodies in patients with pSS and to investigate any associations with their clinical and laboratory characteristics.

## Materials and methods

### Patients

The study involved 155 consecutive SS patients who were evaluated at three tertiary rheumatologic referral centres and who fulfilled the American-European Consensus Group diagnostic criteria [12]. Fourteen patients with SS fulfilling the American College of Rheumatology (ACR) criteria for RA and/or presenting at least one joint erosion [13] were excluded; 141 patients (114 women and 27 men; mean age 48 years, range 39 to 60; mean disease duration 0.98 ± 6.12 years) were considered for the present study. ACR criteria were considered to be fulfilled if at least four criteria were present simultaneously for at least 6 weeks. All of the study subjects had their medical history recorded and underwent a clinical assessment, including 'synovitis' (defined as objective swelling of one or more joints in the absence of erosions) and extra-glandular involvement, erythrocyte sedimentation rate (ESR), C-reactive protein, RF, and anti-CCP, anti-extractable nuclear antigen (ENA), and antinuclear antibodies (ANAs). Hand and foot radiographs were also evaluated. Written informed consent was obtained from the patients before their inclusion in the study, which was approved by the ethics committees of the participating centres. All of the patients with synovitis and anti-CCP antibodies were re-evaluated by an experienced rheumatologist to definitively exclude a diagnosis of RA according to ACR criteria. To be classified as having RA, patients had to have met four criteria simultaneously for at least 6 weeks by the time of the evaluation or in the past.

### Detection of anti-cyclic citrullinated peptide antibodies

Anti-CCP antibodies were tested using a commercially available second-generation enzyme-linked immunosorbent assay (ELISA) kit (Axis-Shield, Dundee, UK) as previously described [14]. The serum samples were evaluated in triplicate, with the upper normal limit of 5 IU/mL being assumed in accordance with the manufacturer's recommendations. Plates from the same batch (#470094) were used in order to avoid any plate-to-plate variations in anti-CCP measurements. Inter- and intra-assay variability was less than 9%.

### Detection of rheumatoid factor

IgM RF was measured by means of immunonephelometry using the quantitative N Latex RF system (Dade Behring, now part of Siemens AG, Munich, Germany). Concentrations of greater than 15 IU/mL were considered positive.

### Detection of antinuclear and anti-extractable nuclear antigen antibodies

ANAs were tested by means of standard indirect immunofluorescence, as previously described [14], using a BX 51 Olympus fluorescence microscope (Olympus Optical Co., Hamburg, Germany) at ×40 power. ENA antibodies were evaluated in triplicate using commercially available ELISA kits (Axis-Shield) according to the manufacturer's recommendations. The following individual ENA specificities were investigated: Sm, RNP, SSA (Ro), SSB (La), Scl-70, and Jo1.

### Radiographic assessment

Radiographs of the hands and feet were evaluated at the time of the anti-CCP analysis. Patients with at least one erosion were excluded from the study.

### Statistical analysis

The data were statistically analysed by means of R software, version 2.5 [15], using the Wilcoxon non-parametric rank sum test for continuous variables, and the Fisher exact or Pearson chi-square test with Yates' continuity correction for discrete variables. All of the analyses were two-tailed. *P *values of less than 0.05 were considered as indicating statistical significance. The variables found to be significantly associated with the prevalence of anti-CCP positivity at the univariate level were examined in a logistic regression model using exact inference (LogXact version 7; Cytel Inc., Cambridge, MA, USA). The prevalence odds ratio (OR) was used as the effect measure in this prevalence study [16].

## Results

Fourteen (9.9%) of the patients with pSS had moderate to high levels of anti-CCP antibody, and 94 (66.7%) were RF-positive, 134 (95%) ANA-positive, 115 (81.6%) SSA-positive, and 55 (39%) SSB-positive (Table 1). The mean value for anti-CCP antibodies was 46 IU/mL (28 to 78), with a normal range (as defined by the manufacturer) of less than 5 IU/mL. None of the 14 patients fulfilled the ACR criteria for RA. The focus score at lip biopsy was greater than 1 in 94 patients (67.1%). Extra-glandular involvement was found in 81 patients (57.4%) (Table 2), and synovitis was found in 44 (31.2%) without any radiographic evidence of erosions (Table 1).

**Table 1:** Demographic and clinical features of 141 patients with primary Sjögren syndrome

	Number	Percentage
Females/Males	114/27	80.9/19.1
Age (range), years	48 (39–60)	
Median disease duration (IQR), years	2.49 (0.98–6.12)	
Synovitis (objective swelling)	44	31.2
Extra-glandular involvement (skin, lung, kidney, or vasculitis)	81	57.4
Median erythrocyte sedimentation rate (IQR), mm/1st hour	27 (17–38)	
Median C-reactive protein (IQR), mg/dL	0.57 (0.31–0.9)	
Rheumatoid factor	94	66.7
Anti-CCP antibodies	14	9.9
Anti-nuclear antibodies	134	95
Anti-La (SSB) antibodies	55	39.1
Anti-Ro (SSA) antibodies	115	81.6
Focus score of greater than 1	94	67.1

Demographic characteristics and continuous data are expressed as median (interquartile range, IQR). Categorical data are expressed as absolute frequency and percentage. Anti-CCP, cyclic citrullinated peptide antibody.

**Table 2:** Extra-glandular involvement in 141 patients with primary Sjögren syndrome subdivided for the specific manifestations

	Number	Percentage
Raynaud phenomenon	81	57
Lymphadenopathy	18	13
Cutaneous vasculitis	9	6
Lung involvement	18	13
Kidney involvement	6	4
Liver involvement	7	5
Peripheral neuropathy	15	11
Myositis	3	2
Lymphoma	0	0

### Rheumatoid factor-positive primary Sjögren syndrome patients

There were no correlations between RF titres and synovitis (*P *= 0.65), extra-glandular involvement (*P *= 0.85), laboratory parameters, or focus scores, and there were no significant differences between the RF-positive and RF-negative patients in terms of age (*P *= 0.26) or disease duration (*P *= 0.55).

### Anti-CCP-positive primary Sjögren syndrome patients

There was a close relationship between anti-CCP positivity and synovitis (*P *< 0.001) but not between anti-CCP positivity and other extra-glandular involvement (*P *= 0.77). There were no significant differences between the anti-CCP-positive and -negative patients in terms of age (*P *= 0.13), disease duration (*P *= 0.41), mean ESR (*P *= 0.10), the presence of anti-SSA (*P *= 0.29), anti-SSB (*P *= 0.78), or RF (*P *= 0.77) or a focus score of greater than 1 (*P *= 0.77) (Table 3).

**Table 3:** Correlations with clinical and laboratory parameters of anti-CCP antibody-positive and -negative primary Sjögren syndrome patients

	Anti-CCP-positive (n = 14)	Anti-CCP-negative (n = 127)	*P *value
Age (range), years	41 (34.5–52.2)	49 (39–60)	0.134
Disease duration, years	2.4 (1.40–7.5)	2.5 (0.98–5.2)	0.415
Synovitis, number (percentage)	12 (85.7)	32 (25.2)	<0.001
Extra-glandular involvement, number (percentage)	9 (64.3)	72 (56.7)	0.777
Erythrocyte sedimentation rate (range), mm/1st hour	34 (24.25–45.5)	27 (16.5–38)	0.101
Rheumatoid factor, number (percentage)	10 (71.4)	84 (66.1)	0.774
Anti-nuclear antibodies, number (percentage)	13 (92.9)	121 (95.3)	0.527
Anti-La (SSB) antibodies, number (percentage)	10 (71.4)	105 (82.7)	0.291
Anti-Ro (SSA) antibodies, number (percentage)	6 (42.9)	49 (38.6)	0.779
Focus score of greater than 1, number (percentage)	9 (64.3)	85 (67.5)	0.773

Anti-CCP, cyclic citrullinated peptide antibody.

### Multivariable analysis of synovitis

An exact logistic regression analysis was used to model the effects of the presence of positive anti-CCP antibodies on the prevalence of synovitis, adjusting for the potential covariates of age, disease duration, gender, and anti-SSA and anti-SSB antibodies. Age and disease duration were divided into quartiles with the aim of simplifying the interpretation of the results by comparing the oldest patients and those with the longest disease duration. Anti-SSA and anti-SSB antibodies were included in the model because they were found in 60% of the patients and are associated with more frequent extra-glandular manifestations [4,17]. As shown in Table 4, multivariate analysis of the anti-CCP-positive patients, adjusted for age, gender, disease duration, and anti-SSA and anti-SSB antibodies, confirmed the association with an increased prevalence of synovitis (prevalence OR versus anti-CCP-negative patients = 7.611, 95% confidence interval [CI] 1.475 to 74.870; *P *= 0.010). None of the covariables included in the model was statistically significant except for anti-SSB antibodies, which were of borderline significance (prevalence OR versus anti-SSB-negative patients = 0.393, 95% CI 0.130 to 0.967; *P *= 0.084). Diagnostic procedures did not reveal any violations of the model assumptions, and the test of the deviance did not provide any evidence requiring the rejection of the null hypothesis of goodness-of-fit (*P *> 0.23).

**Table 4:** Multivariable associations between patient characteristics and the prevalence of synovitis

Model term	OR	95% CI for OR	*P *value
Gender			
Female	1.104	0.279–4.659	1.000
Male	1		
Disease duration, years			
<1	1.772	0.416–8.270	0.576
1 to <2.5	3.926	0.722–25.120	0.133
2.5 to <6	0.908	0.176–4.711	1.000
≥ 6	1		
Age, years			
<40	1.423	0.3217–6.597	0.837
40 to <50	1.307	0.2818–6.179	0.951
50 to <60	1.258	0.205–7.421	1.000
≥ 60	1		
Anti-CCP			
Positive	7.611	1.475–74.870	0.010
Negative	1		
SSA			
Positive	1.588	0.376–7.413	0.691
Negative	1		
SSB			
Positive	0.393	0.130–0.967	0.084
Negative	1		

Anti-CCP, cyclic citrullinated peptide antibody; CI, confidence interval; OR, prevalence odds ratio.

## Discussion

Musculoskeletal manifestations such as fatigue, myalgia, arthralgia, an intermittent non-erosive polyarthropathy affecting mainly the small joints, and usually mild synovitis are common in patients with pSS and may mimic RA, particularly in the presence of RF [2,3,18], although the fact that tests for anti-CCP antibodies usually are negative may help to differentiate the two conditions [19,20].

We found slightly high anti-CCP levels in 14 of our 141 patients with pSS (9.9%). Some studies have found that anti-CCP antibodies are an independent factor predicting the development of erosions in patients with RA [8,9], but none of our 44 pSS patients with synovitis (31.2%) showed any radiographic sign of erosions. After a long follow-up, Gottenberg and colleagues [11] found that 80 out of 134 pSS patients with no radiographic evidence of erosions (59%) were positive for IgM RF, 10 (7.5%) for anti-CCP, 7 (5.2%) for AKA, and 5 (3.7%) for both anti-CCP and AKA.

We also found no significant differences between our anti-CCP-positive and -negative patients in terms of demographic factors or extra-glandular manifestations. However, the presence of synovitis in pSS patients did seem to be closely associated with the presence of anti-CCP antibodies as the odds of having synovitis were 1.475 times greater in our anti-CCP-positive patients, regardless of the other covariables in the model. There were no other significant associations with clinical or laboratory parameters between the anti-CCP-positive and -negative patients.

In addition, anti-SSB positivity was weakly and inversely associated with the prevalence of synovitis. Antibodies to the Ro/SSA and La/SSB ribonucleoprotein particles usually are found in the serum of pSS patients and are associated with a longer disease duration, more frequent non-exocrine manifestations, and more intense lymphocytic infiltration of the minor salivary glands [21]. The prevalence of anti-SSB is higher in pSS, but in our patient cohort it was also inversely related to the presence of synovitis.

The possibility that our anti-CCP patients represent a subgroup of patients with RA and secondary SS was ruled out by excluding patients fulfilling ACR criteria at the beginning of the study and re-evaluating all of the anti-CCP-positive patients. In our study, we decided to be very stringent in the application of ACR criteria, and we classify as RA those patients in whom clinical symptoms (symmetrical arthritis, arthritis of the hands, more than three groups of joints involved, and morning stiffness) were simultaneously present at the moment of evaluation or for at least 6 weeks in the past. In fact, even if we had considered the possibility of a diagnosis of RA for symptoms that were successive and not simultaneously present, none of the 14 patients would have been classifiable as having RA (data not shown).

Indeed, the possibility that patients with anti-CCP antibodies may develop RA cannot be ruled out by the present study, and it is possible that in the future anti-CCP will be interpreted as an additional marker of definite RA. It should be noted, however, that anti-CCP antibodies are strictly associated with the development of erosions in RA and that none of our patients developed erosions after a mean follow-up of 2.4 years.

As a matter of fact, anti-CCP antibodies may also be observed in a minority of patients with other systemic autoimmune diseases. Takasaki and colleagues [22] detected anti-CCP antibodies in patients with mixed connective tissue disease (MCTD) (9%), systemic lupus erythematosus (14%), systemic sclerosis (13%), polymyositis/dermatomyositis (14%), and SS (18%). Some of these patients presented overlapping RA and connective tissue diseases (particularly MCTD) and so, as in the case of other autoimmune diseases, anti-CCP positivity in pSS may be a marker of synovial tissue inflammation. The results of our study suggest that the production of anti-CCP antibodies may be less related to the pathogenesis of RA. It is possible that marked B-lymphocyte hyper-reactivity (a characteristic of pSS) may explain the presence of anti-CCP antibodies, as usually is observed in the case of RF and anti-SSA and anti-SSB antibodies [23].

## Conclusion

Only a minority of patients with pSS are anti-CCP-positive, but the prevalence of synovitis seems to be closely associated with the presence of anti-CCP antibodies as multivariate analysis confirmed the association between anti-CCP positivity and an increased prevalence of synovitis. However, the possibility that patients with anti-CCP antibodies may develop RA cannot be ruled out, particularly in patients with the concomitant presence of IgM RF.

## Abbreviations

ACR = American College of Rheumatology; AKA = anti-keratin antibody; ANA = antinuclear antibody; anti-CCP = cyclic citrullinated peptide antibody; CI = confidence interval; ELISA = enzyme-linked immunosorbent assay; ENA = anti-extractable nuclear antigen; ESR = erythrocyte sedimentation rate; MCTD = mixed connective tissue disease; OR = odds ratio; pSS = primary Sjögren syndrome; RA = rheumatoid arthritis; RF = rheumatoid factor; SS = Sjögren syndrome.

## Competing interests

The authors declare that they have no competing interests.

## Authors' contributions

FA and PS-P contributed to the conception of the study and the acquisition, analysis, and interpretation of data and participated in drafting the manuscript. NL participated in the analysis and interpretation of data. EB and FB-P contributed to the acquisition of data. RC contributed to the interpretation of data and to the critical review of the manuscript. CM provided the final approval of the version of the manuscript to be published. All authors read and approved the final manuscript.
